# Supplementary material for: Longan Polysaccharide as Adjuvant for Cyclophosphamide-Induced Side Effects in Murine Model
Source: Foods. 2025 Aug 21;14(16):2901. doi: 10.3390/foods14162901 (PMC12385366; doi:10.3390/foods14162901)
Supplement: Supplementary file 1 [file foods-14-02901-s001.zip › foods-3772608-supplementary.pdf]

Supporting information

Supplementary figures

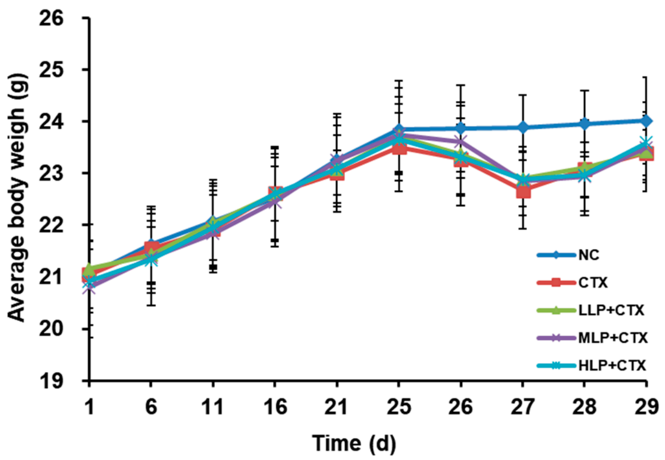

Figure S1 Effect of LP on the body weight of CTX-treatment mice. Error bars represent mean ± SEM (n = 10). Bars sharing different letters indicate $P < 0.05$ by *Tukey's HSD*.

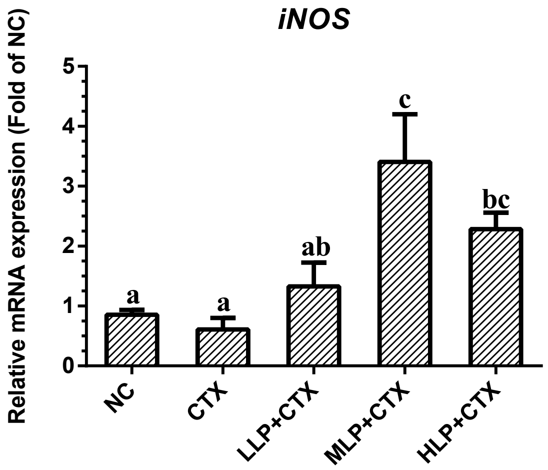

Figure S2 LP administration regulated *inos* mRNA expression in small intestine of CTX-treated mice. Error bars represent mean ± SEM (n = 10). Bars sharing different letters indicate $P < 0.05$ by *Tukey's HSD*.

Supplementary Tables

Table S1 Structural features and immunomodulatory/inti-inflammatory potencies of LP and other polysaccharides.

Polysaccharides	Structure characteristics	Model	Immune modulatory/inti-inflammatory effects	References
Ganoderma atrum polysaccharides	Backbone of 1,3- and 1,6-Glc*p* branched at *O*-3 and *O*-6 with Gal*p* and Glc*p*	CTX-induced immunosuppressed mice	Restore the levels of IL2, IL-10, IgA, IgM and IgG; increase the total antioxidant capacity, activities of superoxidase dismutase, catalase and glutathione peroxidase, and decrease the malondialdehyde level i*n vivo*	[8]
Squid ink polysaccharide	Main glycosidic bonds of -[3GlcA1–4(GalNAca1–3)Fuc1]n-	CTX-induced immunosuppressed mice	Promote secretion of intestinal SIgA	[9]
Purple sweet potato polysaccharides	/	CTX-induced immunosuppressed mice	Enhance the levels of serum TNF-α, IL-2 and IL-6	[10]
Homogalacturonan from *Citrus medica 'fingered'*	Main chain is main chain is composed of →[4)-α-D-GalA*p*-6-O-CH_3(1]–5→3,4)-α-D-GalA*p*-6-O-CH_3	DSS-induced mice	Reduce serum levels of IL-6 and TNF-α	[12]
Polysaccharides from *Hippophae rhamnoides*	High-methoxyl homogalacturonan	CTX-induced immunosuppressed mice	Enhance macrophage production of NO, IL-1β, and IL-6 via the TLR-4/NF-κB pathway	[13]
Arctium lappa L. polysaccharides	Main chain is composed of fructose and glucose, while the branches are galactose and arabinose	*In vitro* intestinal model composed of LPS-stimulated macrophage RAW 264.7 cells and IL-1β-treated colonic Caco-2 cells	Inhibit the production of pro-inflammatory cytokines including IL-8, IL-6, and IL-1β by downregulating the TLR4/NF-κB pathway	[14]

Sulfated Chinese yam polysaccharides	/	LPS-treated Caco-2/RAW264.7 coculture cells	Increase IL-1βand TNF-αby MAPK signaling pathways	[36]
Sugar beet pectin	Main chain consists of alternating rhamnose and galacturonic acid units and is substituted with short neutral side chains of galactose and arabinose	Bone marrow derived dendritic cells in mice	Fermented sugar beet pectin stimulates dendritic cells to produce higher levels of TNF-α and IL-6, as soluble galactose side chain and galacturonic acid skeleton are exposed after fermentation	[37]
Lemon pectin	α-(1-4)-linked galacturonic acid backbone	Caerulien-induced hyperstimulation mice	Suppress intestinal inflammation	[38]
Galactomannan from black soybean hulls	Primarily composed of →4)-β-D-Man*p*-(1→and →4,6)-β-D-Man*p*-(1→, branched with α-D-Gal*p*-(1→, α-D-Gal*p*-(1→6)-α-D-Glc*p*-(1→, and α-L-Ara*f*-(1→at O-6	RAW264.7 macrophages	Increase phagocytosis and boost NO and cytokine release via MAPK and NF-κB signaling	[39]
Longan polysaccharides fraction LPIa and LPIIa	Main glycosidic bonds of →3)-α-Ara*f*-(1→, and →3,6)-β-Gal*p*-(1→, banched side chain of Ara*f*-(1→	LPS-treated Caco-2/RAW264.7 coculture cells	Inhibits TNF-α and IL-8 by downregulating the NF-κB signaling pathway	This study
Longan polysaccharides fraction LPIIIa and LPIVa	Main glycosidic bonds of→3)-α-Ara*f*-(1→, and →3,6)-β-Gal*p*-(1→, banched side chain of →3)-β-Gal*p*A-(1→ and α-Rha*p*-(l→	LPS-treated Caco-2/RAW264.7 coculture cells	Inhibit TNF-α and IL-8 by downregulating TLR4 expression	This study
